# First Virtual International Congress on Cellular and Organismal Stress Responses, November 5–6, 2020

**DOI:** 10.1007/s12192-021-01192-7

**Published:** 2021-02-09

**Authors:** Patricija van Oosten-Hawle, Steven Bergink, Brian Blagg, Jeff Brodsky, Adrienne Edkins, Brian Freeman, Olivier Genest, Linda Hendershot, Harm Kampinga, Jill Johnson, Antonio De Maio, Dan Masison, Kevin Morano, Gabriele Multhoff, Chris Prodromou, Veena Prahlad, Ruth Scherz-Shouval, Anastasia Zhuravleva, Mehdi Mollapour, Andrew W. Truman

**Affiliations:** 1 School of Molecular and Cell Biology and Astbury Centre for Structural Molecular Biology, University of Leeds, Leeds, LS2 9JT UK; 2 Department of Biomedical Sciences of Cells and Systems, University Medical Center Groningen, Groningen, AV 9713 The Netherlands; 3 Department of Chemistry & Biochemistry, College of Science, University of Notre Dame, Notre Dame, IN USA; 4 Department of Biological Sciences, University of Pittsburgh, Pittsburgh, PA USA; 5 Biomedical Biotechnology Research Unit, Department of Biochemistry and Microbiology, Rhodes University, Grahamstown, 6140 South Africa; 6 Centre for Chemico- and Biomedicinal Research, Rhodes University, Grahamstown, 6140 South Africa; 7 Department of Cell and Developmental Biology, University of Illinois Urbana-Champaign, Urbana, IL 61801 USA; 8 Aix Marseille University, CNRS, BIP UMR, 7281 Marseille, France; 9 Department of Tumor Cell Biology, St. Jude Children’s Research Hospital, Memphis, TN 38105 USA; 10 Department of Cell Biology, University of Groningen, University Medical Center Groningen, Ant. Deusinglaan 1, 9713 AV Groningen, The Netherlands; 11 Department of Biological Sciences and the Center for Reproductive Biology, University of Idaho, Moscow, ID 83844 USA; 12 Division of Trauma, Critical Care, Burns and Acute Care Surgery, Department of Surgery, School of Medicine, University of California San Diego, La Jolla, CA 92093 USA; 13 Department of Neurosciences, School of Medicine, University of California San Diego, La Jolla, CA 92093 USA; 14 Laboratory of Biochemistry and Genetics, National Institute of Diabetes and Digestive and Kidney Diseases, National Institutes of Health, 8 Center Dr, Room 324, Bethesda, MD 20892 USA; 15 Department of Microbiology and Molecular Genetics, University of Texas Medical School at Houston, Houston, TX 77030 USA; 16 Radiation Immuno-Oncology Group, Center for Translational Cancer Research (TranslaTUM), Technical University of Munich (TUM), 81675 Munich, Germany; 17 Department of Radiation Oncology, School of Medicine, Technical University of Munich (TUM), 81675 Munich, Germany; 18 Genome Damage and Stability Centre, University of Sussex, Brighton, BN1 9RQ UK; 19 Department of Biology, Aging Mind and Brain Initiative, University of Iowa, Iowa City, IA USA; 20 Iowa Neuroscience Institute, University of Iowa, Iowa City, IA USA; 21 Department of Biomolecular Sciences, The Weizmann Institute of Science, Rehovot, Israel; 22 School of Molecular and Cell Biology and Astbury Centre for Structural Molecular Biology, University of Leeds, Leeds, LS2 9JT UK; 23 Department of Urology, SUNY Upstate Medical University, Syracuse, NY 13210 USA; 24 Department of Biochemistry and Molecular Biology, SUNY Upstate Medical University, Syracuse, NY 13210 USA; 25 Upstate Cancer Center, SUNY Upstate Medical University, Syracuse, NY 13210 USA; 26 Department of Biological Sciences, University of North Carolina at Charlotte, Charlotte, NC 28223 USA

**Keywords:** CSSI Congress, Chaperones, Cancer biology, Heat shock proteins, Hsp70, Hsp90, Proteostasis, Stress responses

## Abstract

Members of the Cell Stress Society International (CSSI), Patricija van Oosten-Hawle (University of Leeds, UK), Mehdi Mollapour (SUNY Upstate Medical University, USA), Andrew Truman (University of North Carolina at Charlotte, USA) organized a new virtual meeting format which took place on November 5–6, 2020. The goal of this congress was to provide an international platform for scientists to exchange data and ideas among the Cell Stress and Chaperones community during the Covid-19 pandemic. Here we will highlight the summary of the meeting and acknowledge those who were honored by the CSSI.

## Introduction

**Fig. 1:**
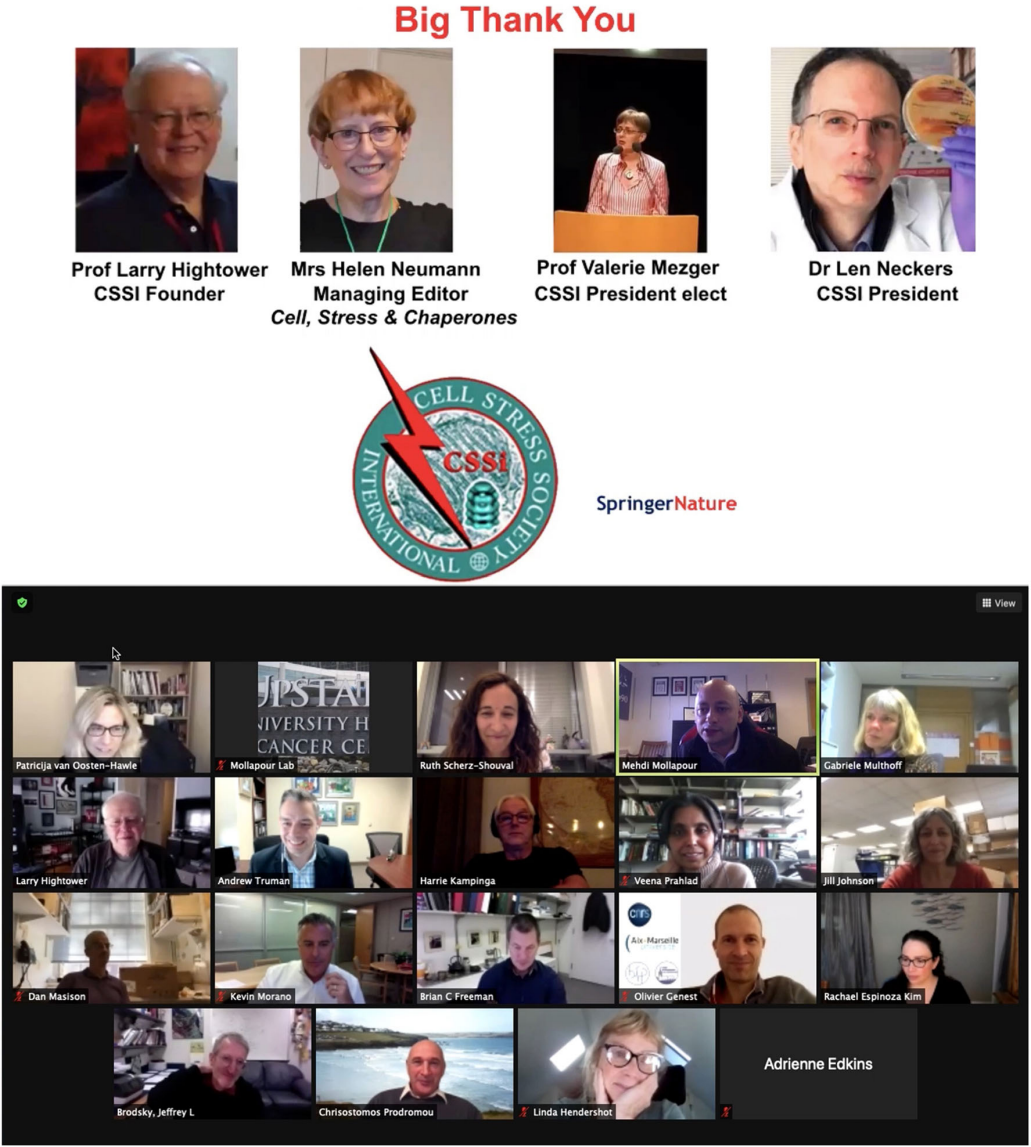
First Virtual International Congress on Cellular and Organismal Stress Responses was held with great success on November 5–6, 2020, with over 300 participants. We thank the senior members of CSSI for creating and maintaining an inspiring and inclusive environment for new and established researchers to thrive

Research on chaperones and the stress response has continued despite the unprecedented disruptions caused by the COVID pandemic. This congress, held virtually in place of the usual Alexandria-Old Town meeting, was attended by over 300 people and brought together diverse speakers from all over the world. Topics of discussion were as varied as the stress response itself and ranged from talks on the fundamental properties of molecular chaperones to the relationship between the stress response and cancer. The program included 4 keynote speakers (Linda Hendershot, Harm Kampinga, Chris Prodromou, and Veena Prahlad) and 13 invited speakers. Larry Hightower (CSSI Founding President) and the principal organizers (Patricija van Oosten-Hawle, Andrew Truman, and Mehdi Mollapour) opened the meeting and introduced Linda Hendershot who gave the Susan Lee Lindquist Science without Boundaries Lecture on “ER mediated maintenance of cellular proteostasis”, Fig. 1.

## The endoplasmic reticulum stress response

The fidelity of cell surfaces and secreted proteomes is dependent not only on the correct maturation of these proteins but also on the ability to detect and destroy proteins that fail to reach their native state. To better understand how these quality control decisions are executed in the endoplasmic reticulum, Linda Hendershot (St. Jude Children’s Research Hospital, USA) explained how her group developed the first in vivo peptide screen to identify binding preferences for the Hsp70 cognate BiP and its co-chaperones that are effectors in these processes. She revealed that sites for pro-folding chaperones BiP and ERdj3 were frequent, dispersed throughout two clients, and consistent with previous in vitro peptide screens. Conversely, pro-degradation co-chaperones Grp170, ERdj4, and ERdj5, which had not been previously queried, recognized a distinct type of sequence that was longer, rich in aromatic residues, and possessed a high aggregation potential. Mutational analyses provided insights into sequence recognition characteristics for these pro-degradation chaperones, which could be readily introduced or disrupted, and the consequences for client fates determined. Her fascinating data revealed unanticipated diversity in recognition sequences for chaperones, established a sequence-encoded interplay between protein folding, aggregation, and degradation, and highlighted the ability of clients to co-evolve with chaperones ensuring quality control.

Similar to other Hsp70s, BiP functions rely on their ability to cycle between several functionally distinct conformations. Anastasia Zhuravleva (University of Leeds, UK) exploited solution NMR and other biophysical techniques to characterize the BiP chaperone cycle and elucidate how the BiP chaperone cycle is regulated post-translationally by AMPylation of the BiP substrate-binding domain (SBD) and binding Ca^2+^ to the BiP nucleotide-binding domain (NBD). Her results suggest that local perturbations in the BiP SBD and NBD fine-tune the pre-existing conformational ensemble, enabling gradual fine-tuning of BiP chaperone activity. These tunable properties of the chaperone cycle for BiP and other Hsp70s potentially provide new opportunities to develop allosteric modulators of their chaperone activity (Wieteska et al. 2017).

Chaperones (both cytoplasmic and ER-localized) are critical in tumor progression and metastasis. Jeff Brodsky (University of Pittsburgh) discussed the ability of cancer cells to withstand severe stress, and suggested that these cells might have been re-wired to withstand these stresses. Expanding upon this concept, his lab identified and used Hsp70 inhibitors to identify cancer cells that are susceptible to proteotoxic stress, and then determined the mechanisms that allow these cells to become resistant to the inhibitors. He then summarized published and new data on the role of the autophagy pathway in mediating resistance.

## Cellular protein quality control

Molecular chaperones are not solo players; their activity and specificity are dictated by a large number of semi redundant co-chaperone proteins. Keynote speaker Harm Kampinga (UMCG, The Netherlands) discussed the peculiar features of the Hsp70 co-chaperone DNAJB6, one of the 50 members of the J-domain protein family. DNAJB6 has excellent properties and capacities to delay the amyloidogenesis of a variety of disease-causing proteins, most particularly those with expanded polyglutamine sequences (polyQ proteins). DNAJB6 expression levels are tightly associated with cellular vulnerability to polyQ aggregation and toxicity and these expression levels have been found to decline upon differentiation from stem cells to neurons, consistent with the neuronal degeneration to which these polyQ proteins lead. DNAJB6 does not interact with polyQ monomers, but with (small) polyQ oligomers when formed and next prevents their transition into amyloid-like aggregates.

Steven Bergink (UMCG, The Netherlands) showed that DNAJB6 is also involved in Nuclear Pore Complex (NPC) biogenesis. Impairment of DNAJB6 results in the accumulation of the so-called annulate lamella—not fully assembled NPCs—in the cytosol. Moreover, DNAJB6 localizes to NPC—assembly intermediates—during the interphase of the cell cycle, and interacts with FG nucleoporins (FG-Nups). The FG-rich domains of Nups form condensates in the cytosol that progress into amyloids over time, a process that is attenuated by DNAJB6. This activity is reminiscent of the reported anti-amyloidogenic action DNAJB6 has on polyglutamine proteins or amyloid-β42. Their data provide the first evidence for a role of chaperones in NPC biogenesis and suggests that DNAJB6 acts as a chaperone for unstructured NPC components, preventing them from aggregating.

Co-chaperone proteins also play an important role in prion propagation. [PSI^+^] prions are amyloids of Sup35, an essential protein. Puzzlingly, [PSI^+^] affects viability only modestly despite substantial depletion of Sup35 into insoluble prion aggregates. Dan Masison (NIH, USA) revealed that [PSI^+^] is however highly toxic to cells with a truncated version of Hsp70 J-protein co-chaperone Sis1. He described a dual role for Sis1 in promoting [PSI^+^] propagation and in keeping Sup35 soluble enough for cells to grow normally. Thus, Sis1 is crucial for [PSI^+^] prions to maintain a balance between depleting enough Sup35 to propagate stably and retaining enough soluble Sup35 for normal growth, which provides an explanation for persistence of otherwise lethal [PSI^+^] prions.

Bacteria represent a huge reservoir of unexplored J-domain proteins (JDPs), the co-chaperones of HSP70/DnaK. By studying the aquatic bacterium Shewanella oneidensis, Olivier Genest (CNRS, Aix Marseille University) revealed that AtcJ, a short JDP of 94 amino acids containing only a J-domain and a C-terminal extremity of 21 amino acids, is required for growth at low temperature. AtcJ interacts through its C-terminal extremity with AtcC, that itself interacts with AtcB, two proteins also important for cold adaptation. Recent results indicate that AtcB contacts the RNA polymerase, suggesting that the Atc proteins could target DnaK to the RNA polymerase (Maillot et al. 2019).

Kevin Morano (University of Texas, USA) presented new work demonstrating that yeast cells defective in redox buffering exhibit chronic activation of a heat shock response. Specifically, mutants in the cytosolic thioredoxin pathway, but not the glutathione or mitochondrial thioredoxin systems, activated the transcription factor Hsf1. Using HSP-GFP fusion proteins, the sequestrate Hsp42 but not the Hsp70/Hsp104 disaggregase machine was found to localize in the perinuclear JUNQ compartment in stable foci. Thioredoxin mutants likewise accumulated the permanently misfolded model protein CPY-GFP but were competent to refold heat-denatured firefly luciferase, suggesting defects in substrate processing through the ubiquitin-proteasome pathway. These findings reveal a previously unknown and important link between redox homeostasis and protein quality control.

## Chaperone structure and function

Over the past several decades, substantial effort has gone into probing the structure and mechanisms of the stress responses. It was exciting to see that there is still room for fundamental discoveries to be made in this area. In his keynote talk, Chrisostomos Prodromou (University of Sussex, UK) presented the cryo-EM structure of the human and yeast R2TP-TTT complex (human: RUVBL1/2-RPAP3-PIH1D1-TELO2-TTI1-TTI2)1. A subcomplex of human R2-TTT (RUVBL1/2) was resolved at 3.4 Å for the R2 (RUVBL1/2) ring and 5 Å for the TTT module. The structures reveal the binding of the TTT complex to the R2 ring. There was evidence that the TP (RPAP3-PIH1D1) component of the R2TP complex was present through tethering, but was essentially not visible, due to the flexible nature of its interaction. Studies using the Kluveromyces maxianus TOR1 showed details of its interaction with the R2TP-TTT complex and which domains were critical for its recruitment (Pal et al. 2020).

Recently, substantial progress has been made in determining direct interactors of Hsp90 (Weidenauer et al. 2017). Adding to this wealth of knowledge, the laboratory of Brian Freeman (UIUC) exploited a number of yeast Hsp90 variants containing the non-natural amino acid p-benzoyl-l-phenylalanine (Bpa), which also serves as a UV-triggered crosslinker, to reveal a broad Hsp90 physical interactome comprised of >1000 hits. Intriguingly, the use of the Bpa-crosslinker identified two different classes of Hsp90 clients—a classic, stably Hsp90-associated one and a more transient Hsp90-regulated class. Validation studies demonstrate that the transient clients depend on Hsp90 in a variety of cellular pathways including transcription, translation, and genome organization.

Similarly, Jill Johnson (University of Idaho) described identification of groups of yeast Hsp90 mutants that have differing effects on Hsp90 interaction with Hsp70 or co chaperones. Mutations within the same group exhibited similar effects on client activity. Surprisingly, one group of Hsp90 mutations had limited effects on activity of select clients, suggesting they result in altered client specificity. Given the large number of PTMs on chaperones, it is possible that some of these mutants are at either sites of modification or in close proximity (Backe et al. 2020; Nitika et al. 2020b).

Adrienne Edkins (Rhodes University, South Africa) described the importance of chaperone interactions in the extracellular matrix. Fibronectin (FN) is a client protein of Hsp90 that is an important component of the extracellular matrix (ECM). Hsp90 interacts via its M-domain directly with N-terminal proteolytic FN fragments, with the binding affinity determined by the intrinsic stability of the FN fragments and the presence of type I FN repeats. Exogenous extracellular Hsp90 altered the size and orientation of individual FN fibers and promoted the incorporation of soluble fibronectin into ECM, consistent with the role of the FN N-terminus in FN ECM assembly.

Chaperones have long been investigated as potential anticancer therapeutic agents, although issues of patient toxicity have remained problematic. Brian Blagg (Notre Dame University, USA) presented preliminary studies that emphasized the development and evaluation of isoform-selective inhibitors of the Hsp90α isoform. Through a structure-based approach small molecules were developed that selectively bound to the Hsp90β isoform in lieu of Hsp90α, which are ~95% identical in the N-terminal ATP-binding sites. A small sub pocket was exposed in Hsp90β that allowed for the inclusion of appendages that when incorporated significantly enhance affinity for Hsp90β, while presenting detrimental interactions with Hsp90α. These new compounds were able to induce the degradation of Hsp90β-dependent substrates in various cell lines and manifested submicromolar EC50s against select cancer cell lines. In addition, no induction of Hsp90 levels was observed, indicating that the Hsp90β-selective inhibitors overcome many of the detriments associated with the pan-Hsp90 inhibitors that were evaluated clinically (Khandelwal et al. 2018).

## Organismal and intercellular level stress responses

Although mechanistic in vitro studies are critical for understanding basic properties of chaperones, it is essential to consider how they are regulated not only at a cellular level but also at an organismal level. Veena Prahlad (University of Iowa) elegantly described neuronal control over the cellular heat shock response in the nematode *C. elegans*. Organisms function despite wide fluctuations in their environment through the maintenance of homeostasis. In general, two distinct kinds of strategies are used by organisms to achieve homeostasis. The first are servomechanisms, whereby error-sensing negative feedback loops triggered by the perturbation of some set-point, correct the performance of a system to restore homeostasis. An alternate mechanism is through the activation of anticipatory or cephalic mechanisms that are predictive and implemented prior to the actual perturbation of the system (Prahlad 2020). Dr. Prahlad discussed work over the last few years from her lab that provides evidence for the existence of a cephalic mechanism of control over the heat shock response. Her data showed that such cephalic control is mediated by the release of the neuromodulator serotonin, linking anxiety, experience, learning—aspects that are fundamental to cephalic processes—to cellular changes in transcription and protein quality control. Finally, she discussed her recent findings that serotonin acts through a signal transduction pathway conserved between *C. elegans* and mammalian cells to enable the transcription factor HSF1 to alter chromatin in soon-to-be fertilized germ cells by recruiting the histone chaperone FACT, displacing histones, and initiating protective gene expression. Without serotonin release by maternal neurons, FACT is not recruited by HSF1 in germ cells and progeny of stressed *C. elegans* mothers fail to complete development (Das et al. 2020). These studies are just beginning to uncover how stress sensing by maternal neurons, coupled to HSF1-dependent transcription in the germline, could result in the epigenetic remodeling of offspring (Das et al. 2020).

HSF1 is also critical in the transcriptional program required for tumor survival. Tumors are stressful environments, and various stress responses are activated in cancer cells and in non-malignant cells of the tumor microenvironment to cope with these stressful conditions. These pathways have been classically shown to be activated in a cell-autonomous manner; however, accumulating evidence over the past years suggests that non-cell-autonomous activation of stress responses plays important roles in tumor progression, metastasis, and immune evasion. Ruth Scherz-Shouval (Weizmann Institute of Science, Israel) showed that heat shock factor 1 (HSF1) is activated in response to inflammatory signals in stromal fibroblasts of the gut, and that its activation promotes ECM remodeling, leading to the development of colon cancer. Loss of HSF1 abrogates ECM assembly by colon fibroblasts in cell culture, prevents ECM remodeling in a mouse model of inflammation-induced colon cancer, and significantly inhibits progression to colon cancer. These findings highlight HSF1 as a key mediator of the response to inflammation in the colon, and highlight another facet of the many roles of stress responses in health and disease (Levi-Galibov et al. 2020).

The chaperone/co-chaperone system is also critical for a robust immune response and cancer cell drug resistance (Nitika et al. 2020a; Shevtsov et al. 2019a). Gabriele Multhoff (Technical University of Munich, Germany) showed that the major stress-inducible Hsp70 (HSPA1A) is frequently overexpressed in the cytosol of a large variety of different tumor entities and presented on the plasma membrane in a tumor-specific manner (Stangl et al. 2018). High cytosolic/membrane Hsp70 levels are associated with therapy resistance and unfavorable prognosis. Moreover, membrane Hsp70 positive tumor cells actively release Hsp70 in exosomes that serve as a biomarker for the membrane Hsp70 status and reflect the viable tumor mass in liquid biopsies (Gunther et al. 2015). Highly aggressive, membrane Hsp70 positive tumor cells can be recognized and killed by Hsp70 peptide TKD and IL-2 (TKD/IL-2) stimulated NK cells in vitro and in vivo. Therefore, patients with non-resectable, advanced NSCLC (stage IIIa/b) were either treated with 4 cycles of ex vivo stimulated NK cells or received best supportive care after radiochemotherapy in a randomized phase II clinical trial. The improved clinical outcome of NSCLC patients after adoptive NK cell transfer was clearly mediated by NK cells expressing activatory (C-type lectin) NK cell receptors. Based on promising preclinical data and a pilot study (Kokowski et al. 2019; Shevtsov et al. 2019b), future plans are to treat NSCLC patients with a combined regimen consisting of ex vivo TKD/IL-2 activated NK cells and immune checkpoint inhibitors in an upcoming clinical trial.

Finally, Antonio De Maio (UC San Diego) discussed Coronavirus Disease 2019 (COVID-19) that is triggered by infection by a new coronavirus, severe acute respiratory syndrome coronavirus 2 (SARS-CoV-2), with a rapid transmission rate (Hightower and Santoro 2020), which has resulted in a worldwide pandemic due to various factors, including propagation from asymptomatic infected individuals, speedy international travel, and poor mitigation approaches adopted in some regions. Although the mortality rate of COVID-19 is less than prior coronavirus epidemics (Fauci et al. 2020), this disease has been a tremendous burden to the world population and economy. The clinical outcome from COVID-19 is modulated by multiple factors, including the level of the infection, the genetic background, gender and age of the patient, and non-genetic factors, such as obesity, smoking, economic status, and environmental conditions. SARS-CoV-2 cellular infection triggers a corresponding activation of the innate immune system that is initially beneficial, but if it is not properly controlled, results in an overwhelming inflammatory response that is detrimental long-term due to the development of innate immune dysfunction, defined as the inability to respond to subsequent stressors, a condition exacerbated by metabolic exhaustion. Therefore, early therapeutic interventions could be critical to ameliorating the outcome from COVID-19. A potential intervention, particularly in conditions of low oxygen saturation levels, is hyperbaric oxygen treatment, consisting of systemic exposure to 100% oxygen under increased atmospheric pressure (De Maio and Hightower 2020; Kjellberg et al. 2020), which appears to be successful in limited clinical trials (Gorenstein et al. 2020; Thibodeaux et al. 2020).

## Awards

The Executive Council of the CSSI has established The Ferruccio Ritossa Early Career Award. The award was created to celebrate the fiftieth anniversary of the discovery of the heat shock response by the late Ferruccio Ritossa who made this pioneering discovery early in his academic career. Len Neckers (CSSI President) presented the 2020 Ritossa Early Career Award to Dimitra Bourboulia (SUNY Upstate Medical University, USA) for her contribution in deciphering the function and regulation of extracellular Hsp90 in cancer. The Executive Council of CSSI has also established the Alfred Tissières Young Investigator Award, in honor and remembrance of Alfred Tissières, a pioneering investigator in the heat shock and cellular stress response field. Larry Hightower (CSSI Founding President) presented the 2020 Alfred Tissières Young Investigator Award to Maxim Shevtsov (Technical University of Munich, Germany). Finally, Larry Hightower presented service awards to the following new CSSI Fellows; Jeff Brodsky, Heath Ecroyd, Adrienne Edkins, Brian Freeman, Pierre Goloubinoff, Patricija van Oosten-Hawle, Jill Johnson, Kevin Morano, Matthias Mayer, Dennis Thiele, Andrew Truman, and Elizabeth Waters. The following were made new CSSI Senior Fellows; Greg Blatch, Wilbert Boelens, Francesco Cappello, Heather Durham, and Cassandra Tierney.

## Concluding remarks

This first virtual meeting was a success, with many new participants all over the world. The virtual nature and free-of-charge registration provided an excellent opportunity to attend the conference and so expanded the CSSI community. It was exciting to see that 17 new members have joined the CSSI since the virtual meeting. Importantly, the conference was attended by many undergraduate researchers with an interest in molecular chaperones and cellular stress responses. CSSI provided free complimentary membership to the society for 1 year to 5 undergraduate researchers who chose to participate at the 2020 virtual conference.

Although virtual meetings can never fully replace in-person meetings that facilitate the formation of new collaborations and wonderful networking opportunities, the great success of this virtual meeting suggests that a hybrid meeting may the format of choice in the years to come.
